# The Book World of Medicine and Science

**Published:** 1901-11-09

**Authors:** 


### The Book World of Medicine and Science.

Surgical Diseases of the Kidney and Ureter, including Injuries, Malformations, and Misplacements. By Henry Morris, M.A., M.B.Lond., F.R.C.S., with two coloured plates and upwards of 200 engravings. In two volumes. (London: Cassell and Co. 1901. Price 42s.)

This is one of the most important works which have been issued during the current year—a complete monograph on the surgical diseases of the kidney and ureter by one who from his experience and his teaching has long been recognised as a master in his art—and we have no doubt that for some time to come it will be regarded as an authoritative book of reference upon the subject with which it deals. What strikes us especially in reading it is its completeness, for while it is evident that the teaching is the outcome of personal experience, it is clear that, before arriving at his conclusions, the author has made a careful study of the works of others. After chapters on the regional anatomy and the abnormalities of the kidney we come to its clinical examination, after which the long series of diseases and accidents to which it is liable, and the operations by which relief for them is sought, are treated seriatim. Then the same course is followed in regard to the ureters. Turning to the much vexed question of movable kidney we find the author speaking favourably of operation in many cases. When, however, a movable kidney is complicated with hysteria, all palliative means should be tried before resorting to an operation, and the patient's friends should be informed of the uncertainty of the result. Statistics show that a cure may be hoped for in about half of these cases. For uncomplicated movable kidney, in which the principal symptoms are pain and gatro-intestinal troubles, the operation may be confidently recommended and carried out without previous trial of belts or rest, while, when renal crises are a feature of the case, nephropexy ought to be strongly urged. Finally, when a movable kidney gives rise to no inconvenience, an operation ought not to be thought of, nor need a belt be worn. The chapters on suppuration and on urinary fever are altogether admirable and contain many useful suggestions as to treatment. In regard to hydronephrosis there is much that is most interesting, the chapters on this subject being illustrated by some remarkable examples of distended and wasted kidneys. The whole subject of renal calculus and its effects is very carefully discussed and it is shown that in the choice of operation the aim should be to extend the application of nephrolithotomy and ureterotomy and thereby restrict the necessity for nephrotomy and nephrectomy. Mr. Morris urges that an unsuspected or quiescent calculus is a source of very real danger, and that when its presence is disclosed we ought to recommend its immediate removal, regardless of the fact that it is not causing pain, unless the condition of the patient contra-indicates an operation. The remarks of Mr. Morris as to the treatment of calculous anuria deserve the most careful consideration. In such cases, as he points out, there is wont to be a period of tolerance in which the patient, although he may not pass a single drop of urine for several days, may walk about, eat, and do mental work with all the appearance of good health. Thus it happens that decisive treatment is apt to be delayed until the onset of the uræmic stage in which death is so immediately imminent that it is too late to give relief. Mr. Morris, however, strongly insists that the time at which to do an operation is as soon as the anuria is established and the diagnosis is made, for nephrotomy is serious only on account of the condition for which it is performed. The operation should be done on the kidney which has most recently become blocked, if there are means for deciding this point, for the other kidney may for a long time have been wasted and useless. Much of the surgery of the kidney and the whole of that of the ureters is quite recent, and we strongly recommend the careful study of this book to surgeons as a guide to operative treatment, and to physicians in order that they may recognise the extent to which their surgical colleagues are now able to give relief in cases which not so many years ago were practically hopeless.

International Directory of Laryngologists and Otologists. Containing Names and Addresses of Practitioners engaged in the Study and Practice of Laryngology and Otology. Compiled by Richard Lake, F.R.C.S. (London: Rebman, Limited. 1901. Price 5s. net.)

We cannot pretend to approve of this little book. It is a dangerous precedent for any group of medical men to allow their names to appear in a directory the publication of which cannot fail to be regarded as an assertion of a claim to some special knowledge superior to that possessed by their fellow practitioners. The price asked for this *brochure* of 124 small pages seems to us to show that it is addressed to those troubled with the question whom to consult, and if we think for a moment what would be the condition of the medical profession if each of the specialties into which it is divided were to issue its own little list, we can hardly fail to see how bad must be the tendency of all such methods of advertising. We are not informed on what principle the names contained in this directory have been selected, but we note that some very well known in connection with the specialties in question do not appear, so that it may well be surmised that many leaders in professional circles disapprove of the publication of this most objectionable list quite as much as we do.

